# Distinct Expression Patterns of Cxcl12 in Mesenchymal Stem Cell Niches of Intact and Injured Rodent Teeth

**DOI:** 10.3390/ijms22063024

**Published:** 2021-03-16

**Authors:** Pierfrancesco Pagella, César Nombela-Arrieta, Thimios A. Mitsiadis

**Affiliations:** 1 Orofacial Development and Regeneration, Institute of Oral Biology, Medical Faculty, University of Zurich, 8032 Zurich, Switzerland; pierfrancesco.pagella@zzm.uzh.ch; 2 Department of Medical Oncology and Hematology, University Hospital of Zurich, 8091 Zurich, Switzerland; Cesar.NombelaArrieta@usz.ch

**Keywords:** tooth, dental pulp, periodontium, *Cxcl12*, Cxcr4, SDF-1, *Cxcl12*-GFP mice, stem cells, stem niche niches, blood vessels

## Abstract

Specific stem cell populations within dental mesenchymal tissues guarantee tooth homeostasis and regeneration throughout life. The decision between renewal and differentiation of stem cells is greatly influenced by interactions with stromal cells and extracellular matrix molecules that form the tissue specific stem cell niches. The *Cxcl12* chemokine is a general marker of stromal cells and plays fundamental roles in the maintenance, mobilization and migration of stem cells. The aim of this study was to exploit *Cxcl12*-GFP transgenic mice to study the expression patterns of *Cxcl12* in putative dental niches of intact and injured teeth. We showed that endothelial and stromal cells expressed *Cxcl12* in the dental pulp tissue of both intact molars and incisors. Isolated non-endothelial *Cxcl12*^+^ dental pulp cells cultured in different conditions in vitro exhibited expression of both adipogenic and osteogenic markers, thus suggesting that these cells possess multipotent fates. Taken together, our results show that *Cxcl12* is widely expressed in intact and injured teeth and highlight its importance as a key component of the various dental mesenchymal stem cell niches.

## 1. Introduction

Tooth is the most mineralized organ of the body that maintains its vitality throughout life thanks to two highly vascularized and innervated cranial neural crest-derived mesenchymal tissues, the dental pulp and the periodontium [1,2]. The dental pulp occupies the central portion of the tooth and contains, between other cell populations, the odontoblasts that synthesize and secrete the organic matrix of dentin. The periodontium occupies the space between the tooth roots and the surrounding alveolar bone and contains the continuously remodeled periodontal ligament that offers stability to the tooth by absorbing the masticatory loads [3,4]. Mesenchymal stem cells (MSCs) located in these two dental components provide teeth with suitable regenerative capabilities in case of traumatic or pathological assaults [5,6,7,8]. To fully exploit their regenerative potential, various dental MSC populations have been isolated, characterized and extensively studied both in vitro and in vivo during the last two decades [6,9,10,11,12]. However, the nature and exact location of these MSC populations are not yet completely known. Stem cell niches form specific microenvironments in defined anatomic locations of each tissue enabling MSCs to survive, self-renew, and change their number and fates [13]. The interactions between stem cells and stromal cells of the niche regulate MSC behavior in tissue maintenance and regeneration [14]. It is therefore necessary to identify cell populations within the dental tissues that are part of the specific microenvironment formed by MSCs and stromal cells in order to better understand tooth biology in homeostatic and regenerative conditions. 

Cxcl12 is a secreted chemokine that plays a major role in the maintenance, mobilization and migration of stem cells [15,16,17,18,19,20]. *Cxcl12* is expressed in many organs and tissues, including bone marrow, brain, heart, liver, lungs, and teeth [16,20,21]. *Cxcl12* is expressed by stromal cells in the bone marrow [22], where it is necessary for the maintenance and homing of hematopoietic stem cells [17,21]. In the brain, Cxcl12 is part of the adult neural stem cell niche [16,23]. Cxcl12 interacts with its receptor Cxcr4, which in turn induces the activation of several intracellular signaling cascades, such as the mitogen-activated protein kinases (MAPK), phospholipase C, and phosphatidylinositol-3-kinase pathways [15,24]. This signaling axis exerts fundamental functions during development and regeneration by affecting cellular migration, adhesion and differentiation [15,24]. Deletion of Cxcl12/Cxcr4 in mice leads to perinatal death due to severe defects in heart and neural development, vascularization, and immune cells maturation [25,26]. In chicken embryos, lack of Cxcl12/Cxcr4 signaling induces severe alterations in neural crest cells migration that lead to craniofacial malformations reminiscent of the DiGeorge syndrome [27]. During regeneration Cxcl12 acts as a strong chemoattractant for stem cells thus promoting tissue healing [19,20,28,29,30,31]. However, Cxcl12 does not act exclusively on MSCs, but it is also involved in neurogenesis [32,33,34], angiogenesis [35,36,37], and immune system function [18]. Studies in teeth have shown that *Cxcl12* expression is upregulated in the dental pulp and periodontium upon inflammation and injury [31,38,39]. Cxcl12 is actively involved in the migration of MSCs and promotes neo-angiogenesis during the regeneration of these two dental tissues [40,41,42,43,44,45,46,47].

Although Cxcl12 has been deeply studied in tooth pathology and repair, there is patchy and little or no information concerning its exact localization and its eventual participation in the composition of stem cell niches in dental tissues of intact adult teeth [31,38]. With the aim to address this issue, we analyzed the expression of *Cxcl12* in molars and incisors of adult *Cxcl12*-GFP transgenic mice. We compared the expression of *Cxcl12* with the expression of molecules that characterize the various cell types composing the dental pulp, such as stem cells/progenitor cells, fibroblasts, immune cells, endothelial cells, and neuronal cells. Furthermore, we analyzed the in vivo distribution of the Cxcl12 molecule in the dental pulp upon tooth injury. Finally, we studied the differentiation potential of isolated *Cxcl12*-expressing dental pulp cells in vitro.

## 2. Results

**Figure 1:**
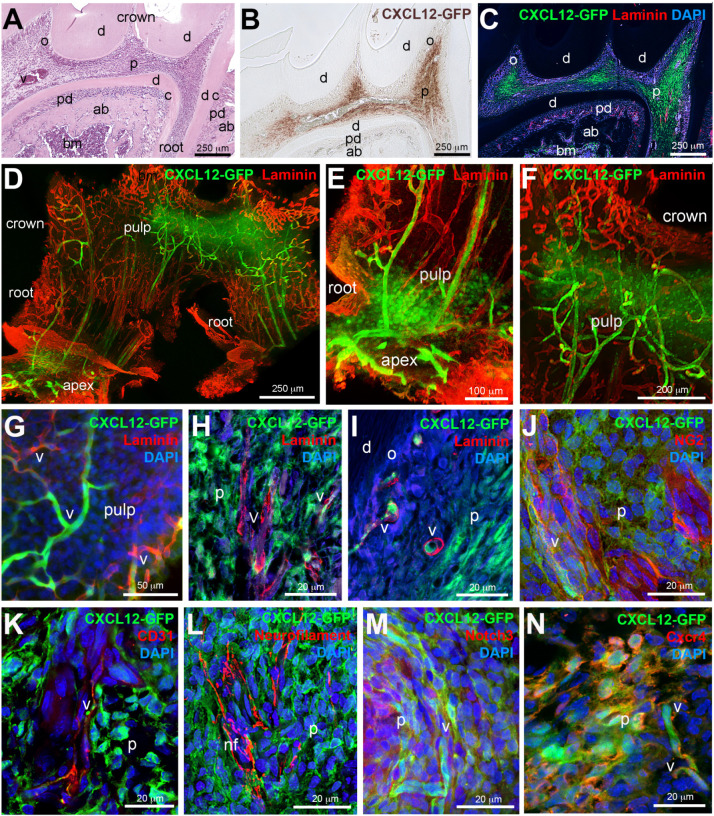
Expression of *Cxcl12* in the first molars from *Cxcl12*-GFP mice. (**A**) Histology of a first mouse molar upon staining with hematoxylin-eosin. (**B**) Immunohistochemistry against *Cxcl12*-GFP (brown color). (**C**) Double immunofluorescent staining against *Cxcl12*-GFP (green color) and Laminin (red color). Cell nuclei in blue color upon DAPI treatment. (**D**) Whole mount immunofluorescent staining against *Cxcl12*-GFP (green color) and Laminin (red color) on the first molar pulp isolated from *Cxcl12-*GFP mouse pups. (**E**,**F**) Higher magnifications of Figure 1D; (**E**) shows the pulp at the tooth root apex, (**F**) shows the pulp corresponding to the tooth crown part. (**G**) Whole mount immunofluorescent staining against *Cxcl12*-GFP (green color) and Laminin (red color) of a dental pulp. Cell nuclei in blue color upon DAPI treatment. (**H**,**I**) Immunofluorescent staining against GFP (green color) and Laminin (red color), showing the localization of *Cxcl12*-GFP^+^ cells relative to blood vessels (marked by Laminin expression). Cell nuclei in blue color upon DAPI treatment. (**H**) shows the central core of the pulp, where *Cxcl12*-GFP^+^ cells are abundantly present. (**I**) shows the margins of the pulp containing the odontoblasts, where *Cxcl12*-GFP^+^ cells are restricted to the blood vessels lumen. (**J**) Immunofluorescent staining against GFP (green color) and NG2 (a marker of pericytes/perivascular mesenchymal stem cells; red color). Cell nuclei in blue color upon DAPI treatment. (**K**) Immunofluorescent staining against GFP (green color) and CD31 (a marker of endothelial cells; red color). Blue color: DAPI. (**L**) Double immunofluorescent staining against GFP (green color) and Neurofilament (red color), showing the localization of *Cxcl12*-GFP^+^ cells relative to neurons (marked by Neurofilament expression). DAPI in blue color. (**M**) Immunofluorescent staining against GFP (green color) and Notch3 (a marker of perivascular mesenchymal stem cells; red color). DAPI in blue color. (**N**) Immunofluorescent staining against GFP (green color) and the Cxcr4 receptor (red color). DAPI in blue color. Abbreviations: ab, alveolar bone; bm, bone marrow; c, cementum; d, dentin; nf, nerve fibers; o, odontoblasts; p, dental pulp; pd, periodontium; v, vessels. Scale bars: (**A**–**D**), 250 μm; (**E**), 100 μm; (**F**), 200 μm; (**G**), 50 μm; (**H**–**N**), 20 μm.

We first determined the distribution of *Cxcl12*-expressing cells in the dental pulp of the molars (Figure 1). We performed immunostaining against GFP on tissue sections obtained from *Cxcl12*-GFP mice (Figure 1B–C). GFP signal was detected in a large area within the dental pulp of molars, both in proximity to vessels and in the core of the pulp (Figure 1B). To obtain a more thorough understanding of the distribution of *Cxcl12*-GFP^+^ cells within the dental pulp, we analyzed dental pulps isolated from *Cxcl12*-GFP mice by whole mount immunofluorescent staining followed by three-dimensional (3D) confocal microscopy imaging (Figure 1D–G). *Cxcl12*-GFP signal was detected in large areas of the dental pulp (Figure 1D), and whole mount immunofluorescent co-staining against GFP and Laminin revealed that *Cxcl12*-GFP^+^ cells were in part localized within the lumen of Laminin^+^ blood vessels (Figure 1D–F) that spanned from the root apex (Figure 1E) to the pulp chamber (Figure 1F). Notably, only a subset of blood vessels showed *Cxcl12*-GFP^+^ cells in their lumen (Figure 1E–G). *Cxcl12*-GFP^+^ cells were also observed outside blood vessels (Figure 1D), and these were clustered at the root apex (Figure 1E) and the central part of the pulp (Figure 1F).

Tissue sections confirmed the abundance of *Cxcl12*-GFP^+^ cells in the core of the pulp (Figure 1H). By contrast, in the margins of the pulp that contain the odontoblasts, the *Cxcl12*-GFP staining was restricted to cells localized at the lumen of the vessels (Figure 1I). 

To analyze the relationship of *Cxcl12*-GFP^+^ cells (green color) with other dental pulp cell populations (in red color), we performed additional immunofluorescent staining against NG2, Notch3 (used as markers for mesenchymal stem cells), CD31 (a marker for endothelial cells), and Neurofilament (a marker for neurons), as well as against Cxcr4, the Cxcl12 receptor (Figure 1J–N). NG2 and Notch3 are expressed by stem cells localized in perivascular niches [48,49]. We detected very few *Cxcl12*-GFP^+^ cells co-expressing MSC markers (having an orange/yellow color). *Cxcl12*-GFP^+^ and NG2^+^ cells were however often observed in neighboring cells associated with vascular structures (Figure 1J). *Cxcl12*-GFP^+^ cells were also observed in close contact with Notch3-expressing perivascular MSC cells (Figure 1M). Double staining against *Cxcl12*-GFP and CD31 indicated that only a minor subset of endothelial cells co-express *Cxcl12*-GFP (orange/yellow color; Figure 1K). Immunostaining against GFP and Neurofilament showed that nerve fibers are in close contact with *Cxcl12*-GFP^+^ pulp cells (Figure 1L). Cxcr4 staining was often localized in close proximity *Cxcl12*-GFP^+^ cells (Figure 1N). However, cells expressing both *Cxcl12*-GFP and Cxcr4 staining (orange/yellow color) were also observed in the dental pulp (Figure 1N). 

**Figure 2:**
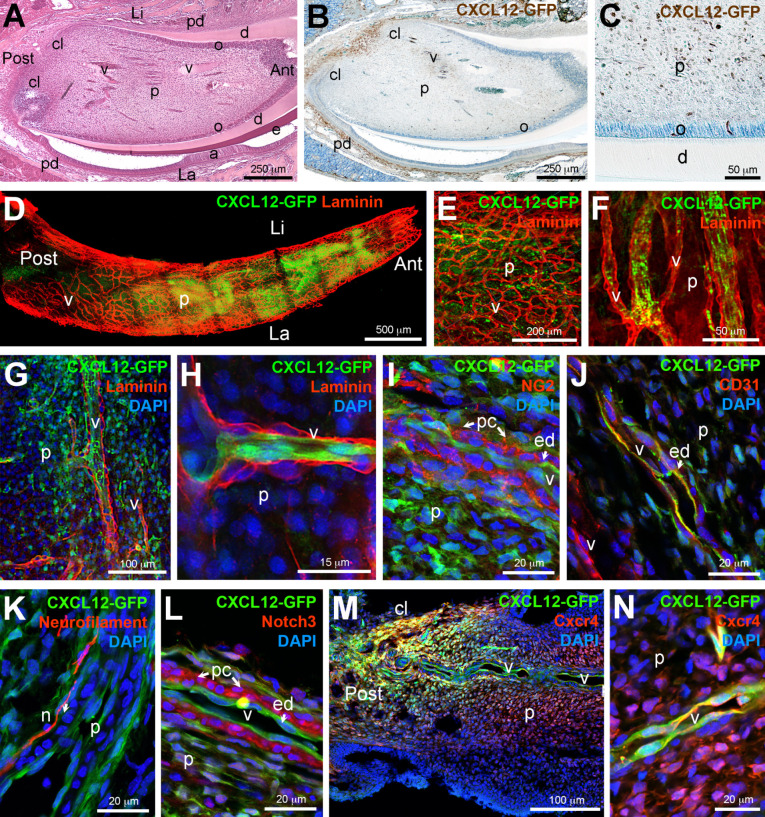
Expression of *Cxcl12* in incisors of *Cxcl12*-GFP mice. (**A**) Histology of a mouse incisor upon staining with hematoxylin-eosin. (**B**) Immunohistochemistry against *Cxcl12*-GFP (brown color). (**C**) Higher magnification of Figure 2B, showing *Cxcl12*-GFP expression in the incisor dental pulp. (**D**) Whole mount immunofluorescent staining against *Cxcl12*-GFP (green color) and Laminin (red color) on the lower incisor pulp isolated from *Cxcl12-*GFP mouse pups. (**E**,**F**) Higher magnifications of Figure 2D; (**E**) shows the presence of numerous *Cxcl12*-GFP^+^ cells in the dental pulp, (**F**) shows circulating *Cxcl12*-GFP^+^ cells in the lumen of blood vessels. (**G**) Whole mount immunofluorescent staining against *Cxcl12*-GFP (green color) and Laminin (red color) in the dental pulp of incisors. Cell nuclei in blue color upon DAPI treatment. (**H**) Higher magnification of Figure 2G. Notice the presence of *Cxcl12*-GFP cells within blood vessels. (**I**) Immunofluorescent staining against GFP (green color) and NG2 (red color). Cell nuclei in blue color upon DAPI treatment. NG2 is expressed by perivascular MSCs (pericytes) (pc; arrows), while adjacent endothelial cells (ed) express *Cxcl12*-GFP (arrows). (**J**) Immunofluorescent staining against GFP (green color) and CD31 (red color). DAPI is in blue color. Notice the presence of *Cxcl12*-GFP^+^ endothelial cells (arrow). (**K**) Double immunofluorescent staining against GFP (green color) and Neurofilament (red color), showing the localization of Cxcl12-GFP^+^ cells relative to neurons. DAPI in blue color. (**L**) Immunofluorescent staining against GFP (green color) and Notch3 (red color). DAPI in blue color. Notice that Notch3-expressing pericytes (arrows) are immediately adjacent to *Cxcl12*-GFP-expressing endothelial cells (arrow). (**M**) Immunofluorescent staining against GFP (green color) and the Cxcr4 receptor (red color). DAPI in blue color. (**N**) Higher magnification of Figure 2M, showing expression of Cxcr4 and *Cxcl12*-GFP in neighboring cells in vessels. Abbreviations: a, ameloblasts; Ant, anterior; cl, cervical loop; d, dentin; e, enamel; ed, endothelial cells; La, labial; Li, lingual; n, nerves; o, odontoblasts; p, dental pulp; pc, pericytes; pd, periodontium; Post, posterior; v, vessels. Scale bars: (**A**,**B**) 250 μm; (**C**,**F**), 50 μm; (**D**), 500 μm; (**E**), 200 μm; (**G**,**M**), 100 μm; (**H**), 15 μm; (**I**–**L**,**N**), 20 μm.

We then characterized the distribution of *Cxcl12*-GFP^+^ cells in the continuously growing mouse incisors (Figure 2A). GFP signal was particularly intense at the posterior part of the pulp of the incisors, in the region located between the epithelial cervical loops (Figure 2B), where stem cells reside. *Cxcl12*-GFP^+^ cells were also found scattered throughout the pulp of the incisors (Figure 2C). *Cxcl12*-GFP signal marked several capillary structures invading the odontoblastic layer (Figure 2C). For better understanding the distribution of *Cxcl12*-GFP^+^ cells within other dental pulp cell populations of the incisors, we analyzed isolated dental pulps from *Cxcl12*-GFP mice by whole mount immunofluorescent staining and three-dimensional (3D) confocal microscopy imaging (Figure 2D–H). Whole mount immunofluorescent co-staining against GFP and Laminin showed that *Cxcl12*-GFP^+^ cells were widely distributed in the dental pulp (Figure 2D). *Cxcl12*-GFP^+^ cells were localized both in the core of the pulp (Figure 2E–G) and in blood vessels (Figure 2F–H). We observed vessels characterized by a high number of *Cxcl12*-GFP^+^ cells and vessels that did not contain *Cxcl12*-GFP^+^ cells (Figure 2F–H). In *Cxcl12*-GFP^+^ vessels, the pericytes were negative for *Cxcl12*-GFP, while the GFP signal was detected in endothelial cells (Figure 2H). 

We further analyzed the relationship of *Cxcl12*-GFP^+^ cells (green color) with other dental pulp cell populations. For this purpose, we performed additional immunofluorescent staining against GFP (marked by green color), NG2, Notch3, CD31, Neurofilament, as well as against Cxcr4 (all marked by red color; Figure 2I–N). Co-staining for GFP and NG2 revealed frequent and direct contacts between *Cxcl12*-GFP^+^ and NG2^+^ perivascular cells, along capillaries and larger vessels located in the core of the pulp (Figure 2I). Co-staining against GFP and CD31 showed that *Cxcl12* was expressed by a subpopulation of endothelial cells. It is obvious from the staining that in the core of the pulp, some blood vessels were completely negative for *Cxcl12*-GFP, while other vessels were formed by a combination of *Cxcl12*-GFP positive and negative endothelial cells (Figure 2J). Non-endothelial *Cxcl12*-GFP^+^ cells could be observed in contact with CD31-positive endothelial cells (Figure 2J). Co-staining for GFP and Neurofilament revealed a close association of *Cxcl12*-GFP^+^ cells with nerve fibers (Figure 2K). No double positive GFP^+^/Neurofilament^+^ cells were detected, indicating that *Cxcl12*-GFP^+^ cells do not have a neuronal identity. Co-staining for GFP and Notch3 showed close contacts between *Cxcl12*-GFP^+^ cells and Notch3^+^ perivascular MSCs (Figure 2L). Notch3^+^ cells were surrounded by *Cxcl12*-GFP^+^ cells from the endothelium and the dental pulp tissue (Figure 2L). Double staining for GFP and Cxcr4 showed a strong correlation between *Cxcl12* and Cxcr4 at the posterior end of the incisors pulp, where MSCs reside (Figure 2M). Cxcr4 was also expressed by perivascular MSCs immediately adjacent to *Cxcl12*-GFP^+^ endothelial cells (Figure 2N). 

We investigated then the expression of Cxcl12 and Cxcr4 in the periodontium (Supplementary Figure S1). In the periodontium (Supplementary Figure S1A), we observed localized expression of *Cxcl12*-GFP (Supplementary Figure S1B–D). *Cxcl12*-GFP was highly expressed in the periosteum lining the alveolar bone and in proximity to the cementum, (Supplementary Figure S1C,D). Double immunofluorescent staining against GFP and Laminin showed close association between *Cxcl12*-GFP^+^ cells and Laminin^+^ blood vessels (Supplementary Figure S1E). Moreover, staining against the Cxcl12 protein was particularly enriched in proximity to the cementum and alveolar bone (periosteum; Supplementary Figure S1F). Labeling against the Cxcr4 receptor was evident in proximity to root cementum and alveolar bone, as well as in perivascular niches (Supplementary Figure S1G).

**Figure 3:**
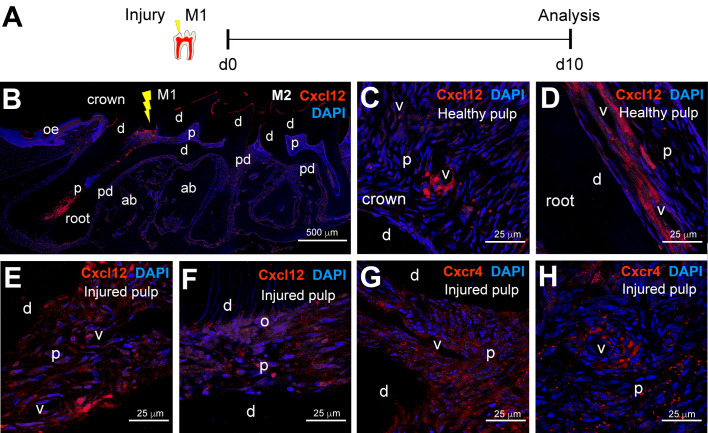
Distribution of Cxcl12 and Cxcr4 molecules in injured dental pulps. (**A**) Experimental design. (**B**) Immunofluorescent staining showing an overview of Cxcl12 protein distribution (red color) upon injury in molars. DAPI in blue color. The yellow arrow marks the injury site. (**C**,**D**) Immunofluorescent staining in dental pulp of intact healthy teeth showing Cxcl12 distribution (red color) in the crown (**C**) and the root region (**D**). DAPI in blue color. (**E**,**F**) Immunofluorescent staining showing Cxcl12 distribution (red color) in injured dental pulp; (**E**) shows a region of the injured pulp distant from the injury site; (**F**) shows Cxcl12 distribution in immediate proximity to the injury. (**G**,**H**) Immunofluorescent staining showing Cxcr4 distribution in injured dental pulp. Abbreviations: ab, alveolar bone; d, dentin; M1, first molar; M2, second molar; o, odontoblasts; oe, oral epithelium; p, dental pulp; pd, periodontium; v, vessels. Scale bars: (**B**), 500 μm; (**C**–**H**), 25 μm.

The distribution of Cxcl12 and Cxcr4 molecules in dental tissues suggests important roles for this signaling axis in the functioning of MSC niches. Therefore, we investigated the distribution of Cxcl12 and Cxcr4 proteins after tooth injury (Figure 3). We performed cavity preparations in molar teeth of adult mice and analyzed their distribution 10 days post-surgery (Figure 3A,B). In the healthy dental pulp, Cxcl12 protein was mostly localized in perivascular areas, adjacent to endothelial cells, both in the crown and in the root (Figure 3C,D). In the injured dental pulp, Cxcl12 protein was more widely expressed, both by cells surrounding the vessels, as well as by cells scattered throughout the dental pulp (Figure 3E). In proximity to the injury, most cells expressed high levels of Cxcl12, including odontoblasts (Figure 3F). The Cxcr4 receptor was expressed by numerous cells within the injured dental pulp (Figure 3G,H). Similar to Cxcl12, Cxcr4-positive cells were localized both around blood vessels (Figure 3H) and sparsely in the dental pulp (Figure 3G).

**Figure 4:**
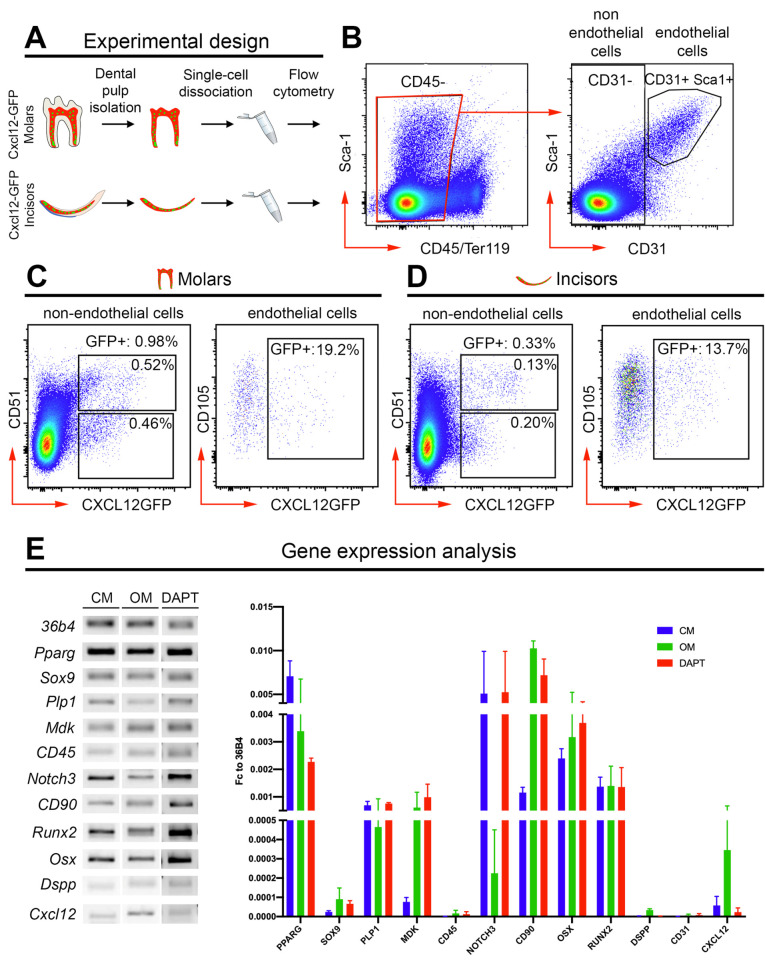
Isolation and characterization of *Cxcl12*-GFP dental pulp cells. (**A**) Experimental design. (**B**) Isolation of nonimmune (CD45^-^) dental pulp cells and sorting of endothelial (CD31+) vs. nonendothelial (CD31^-^) dental pulp cells. (**C**) Fluorescence Activated Cell-Sorter (FACS) quantification of endothelial and nonendothelial Cxcl12-GFP+ cells in molar dental pulps. (**D**) FACS quantification of endothelial and nonendothelial *Cxcl12*-GFP+ cells in incisor dental pulps. (**E**) Gene expression analysis of nonendothelial (CD31^-^) *Cxcl12*-GFP+ dental pulp cells cultured in control medium (CM), osteogenic medium (OM), and in the presence of the Notch pathway inhibitor DAPT (DAPT). Gene expression data obtained from three independent biological replicates.

Thereafter, we further characterized *Cxcl12*-GFP^+^ dental pulp cells by flow cytometry. For this purpose, we isolated dental pulps from molars and incisors of *Cxcl12*-GFP^+^ mice, obtained single cell suspensions, and stained them with markers for immune cells (CD45) and endothelial cells (CD31, Sca1) (Figure 4A,B). We selected CD45^-^ (nonimmune) cells, and sorted CD31^+^ endothelial cells versus CD31^-^ nonendothelial cells (Figure 4B). CD31^+^ and CD31^-^ cells were further analyzed for *Cxcl12*-GFP expression (Figure 4C,D). We observed that only a very minor proportion of CD31^-^, nonendothelial pulp cells expressed *Cxcl12*-GFP both in molars and incisors (0.98% in molars, 0.33% in incisors). A much more abundant proportion of CD31^+^ endothelial cells expressed *Cxcl12*-GFP, both in molars and incisors (19% in molar, 14% in incisors) (Figure 4C,D). 

We then isolated and cultured CD31^-^/*Cxcl12*-GFP^+^ dental pulp cells and characterized their gene expression and differentiation potential in vitro. CD31^-^/*Cxcl12*-GFP^+^ dental pulp cells expressed the dental mesenchymal stem cell markers *Notch3* and *Thy1*/*CD90* (Figure 4E). At the same time, they expressed high levels of the adipogenic differentiation marker *Pparg* and of the osteogenic/odontoblastic differentiation markers *Osx/Sp7* and *Runx2,* as well as detectable levels of the glial marker *Plp1* (Figure 4E). In contrast, we observed very little expression of the odontoblastic differentiation marker *Dspp* (Figure 4E). We also detected very low expression of the chondrogenic differentiation marker *Sox9*, the immune lineage marker *CD45/Ptprc,* and the endothelial cell marker *CD31*/*Pecam1* (Figure 4E). Notably, *Cxcl12* expression was very low in cultured CD31^-^/*Cxcl12*-GFP^+^ dental pulp cells (Figure 4E). 

We then cultured sorted CD31^-^/*Cxcl12*-GFP^+^ dental pulp cells in osteogenic differentiation medium for 10 days and assessed their gene expression. Incubation in osteogenic medium induced opposite effects on the expression of the two mesenchymal stem cell markers *Notch3* and *CD90/Thy1*. *Notch3* was downregulated, while *CD90/Thy1* was strongly upregulated (Figure 4E). We did not observe detectable alterations in the expression of the osteogenic and odontoblastic differentiation markers *Runx2*, and *Dspp*, while *Osx* expression was mildly upregulated (Figure 4E). We observed an increase in the expression of *Mdk*, the gene coding for Midkine, a protein associated with odontoblastic differentiation and necessary for dentin formation [50,51]. We detected a minor decrease in the expression of the adipogenic differentiation marker *Pparg*, although its expression level remained similar to that of the osteogenic differentiation markers *Osx* and *Runx2* (Figure 4E). Culture in osteogenic medium induced a detectable increase in the expression of *Cxcl12* (Figure 4E). 

Since the Notch3 stem cell marker is highly expressed in these cells, we wish to know how the inhibition of the Notch signaling pathway affects the fate of CD31^-^/*Cxcl12*-GFP^+^ dental pulp cells. For this purpose, we cultured CD31^-^/*Cxcl12*-GFP^+^ dental pulp cells in the presence of DAPT, a small molecule that prevents the cleavage of Notch receptors and the subsequent activation of the Notch signaling pathway [52] and assessed their gene expression. Inhibition of Notch signaling did not induce relevant changes in the expression of Notch3 (Figure 4E), while it led to upregulation of *CD90/Thy1* (Figure 4E). The effects of Notch inhibition on the expression of osteogenic, dentinogenic and adipogenic differentiation markers were similar to those observed upon culture in osteogenic medium. DAPT treatment did not cause striking alterations in the expression of the osteogenic markers *Runx2* and *Dspp*, while it induced upregulation of *Osx*, more pronounced than what observed upon culture in the osteogenic medium (Figure 4E). We also observed a strong upregulation in the expression of *Mdk*, and downregulation of the expression of the adipogenic marker *Pparg* (Figure 4E). DAPT treatment did not lead to an upregulation of *Cxcl12* expression (Figure 4E).

## 3. Discussion

Teeth contain a variety of mesenchymal stem cell (MSC) populations that provide them with regenerative potential in case of bacterial or traumatic injury assaults [11]. Specific stem cell niches enable MSCs to survive, self-renew, change their number and fates, and participate in tissue repair [5,13]. The study of the mechanisms that modulate MSC fate and behavior is of paramount importance for understanding the biology of dental tissue homeostasis and regeneration. Although a number of studies in injured and pathological teeth have indicated the important role of Cxcl12 on MSC recruitment, differentiation [29,53,54], as well as on angiogenesis [35], very little and fragmentary information exists concerning the exact location of Cxcl12 in the various stem cell niches of the adult intact and injured teeth [31,38,46]. Our results show that *Cxcl12* is abundantly expressed within the dental pulp, and to a lesser degree in the periodontium, of both molars and incisors. In molars, *Cxcl12* expression is concentrated in perivascular areas and the tooth root apex, both considered MSC niches [55,56]. Perivascular niches are known to contain MSCs that contribute to dental pulp homeostasis and regeneration [48,57,58]. In addition, previous studies have shown that the apical root region of the molar pulp hosts MSCs, which are activated and recruited upon tooth injury [41,55]. Similar to molars, in incisors, *Cxcl12* expression is restricted to perivascular regions and the posterior part of the pulp, where MSCs reside and ensure the continuous growth and remodeling of these teeth [59,60,61]. The localization of *Cxcl12*-GFP-expressing cells at these areas strongly supports an important role for Cxcl12 in the composition of the dental MSC niches. By three-dimensional (3D) whole mount imaging and immunostaining, we show that a proportion of *Cxcl12*-expressing cells constitutes a subset of endothelial cells, while the vast majority of them are non-endothelial cells types that compose the dental pulp. Endothelial *Cxcl12*-expressing cells can represent an important component of perivascular stem cell niches and exert important functions in the modulation of dental MSC behavior [62,63]. Non-endothelial *Cxcl12*-expressing cells are mostly concentrated in the core of the dental pulp and do not express markers for MSCs, neuronal cells, or immune cells, pointing to their identification as dental pulp fibroblasts. Although teeth contain a significant number of MSCs, severe injuries that require higher numbers of MSCs for tissue repair might stimulate the stem cell potential in cells that in normal conditions are not considered stem cells, such as the fibroblasts [64]. Niche-derived signals influence the behavior of dental MSCs, where signaling pathways, such as the Notch pathway, are important regulators of their function [65]. *Cxcl12*-expressing cells are in direct contact with perivascular MSCs that express the stem cell markers Notch3 and NG2 [48,49,58,66,67], as well as the Cxcl12-receptor Cxcr4. These results suggest that Cxcl12 could be directly involved in the regulation of MSCs behavior in dental tissues. This hypothesis is supported by results on injured teeth where Cxcl12 is broadly expressed in dental pulp cells and in odontoblasts. This is in accordance with previous studies that have shown that Cxcl12 is an important molecule for dental tissue repair after various tooth assaults, since its expression is upregulated in dental tissues upon inflammation [31] and Cxcl12 promotes the differentiation of dental pulp MSCs into odontoblasts [68,69]. It is thus conceivable that under the influence of Cxcl12, dental pulp MSCs expressing Cxcr4 migrate from nearby or distant niches to the decay area, where they will engraft and differentiate into new odontoblasts that will form reparative dentin [70,71]. This has been already shown in other organ systems, such as in the case of bone marrow-stem cells transplanted upon myocardial infarction [72,73]. Moreover, Cxcl12 has been shown to promote neovascularization and angiogenesis. 

Flow cytometry analysis shows that, upon dental pulp dissociation, only a tiny percentage of the isolated nonendothelial cells express *Cxcl12*-GFP, in clear contrast with the broad *Cxcl12* expression observed in dental pulp by 3D whole mount imaging and immunostaining. This is in accordance with previous studies conducted on the bone marrow, which showed that flow cytometry quantification of non-endothelial *Cxcl12*-GFP-expressing cells after dissociation detected 30-times less cells compared to 3D whole-mount imaging [22]. Sorted non-endothelial *Cxcl12-*GFP^+^ cells display a low proliferation rate, expression of the MSC markers *Notch3* and *CD90/Thy1*, and high levels of both adipogenic (*Pparg*) and osteogenic (*Runx2*, *Osx*) differentiation markers in vitro. This expression pattern suggests that the few *Cxcl12-*GFP-expressing cells isolated by flow cytometry could represent a tiny population of *Cxcl12-*GFP-expressing dental pulp MSCs or progenitor cells. Culture of non-endothelial *Cxcl12-*GFP-expressing cells in osteogenic conditions led to the upregulation of *Cxcl12* and *Mdk,* a gene associated with odontoblastic differentiation and necessary for dentin formation [50,51]. No relevant changes were however observed in the expression of the osteogenic differentiation markers *Runx2* and *Osx*, which code for two transcription factors necessary for osteoblastic differentiation [74,75]. The osteogenic medium induced opposite effects on the two MSC markers analyzed, as it downregulated *Notch3* expression and upregulated *CD90/Thy1* expression. These cells thus seem to possess multilineage differentiation potential, although less pronounced when compared to unsorted mouse dental pulp MSCs [76]. Notably, the pharmacological inhibition of Notch signaling in *Cxcl12-*GFP-expressing cells had similar effects to those observed upon their treatment with osteogenic medium, in accordance with previous reports showing that Notch signaling inhibits osteogenic differentiation of dental pulp MSCs [77]. This result thus indicates that Cxcl12-GFP-expressing cells are responsive to Notch signaling modulation. 

The periodontium, root cementum and alveolar bone are tissues characterized by constant and intense remodeling, associated with new extracellular matrix formation, cell differentiation and mineral deposition [3,4]. *Cxcl12* expression in the periodontium suggests a possible involvement of this molecule in modulating MSC behavior within periodontal perivascular niches and regulating their differentiation in proximity to the root cementum and alveolar bone. Many periodontal cells express both Cxcl12 and the Cxcr4 receptor, suggesting that Cxcl12/Cxcr4 signaling could be a common modulator of MSC activity within the dental pulp and periodontium. Accordingly, previous works showed that Cxcl12 mediates the recruitment of bone marrow-derived stem cells [39], promoting their differentiation upon periodontal injury [45,78,79,80]. Moreover, overexpression of Cxcl12 in periodontium has been shown to promote neovascularization and angiogenesis [81]. 

In conclusion, our results show the precise expression patterns of Cxcl12 in mesenchymal tissues of intact and injured teeth and highlight the importance of this molecule as a key component of the dental MSC niches. 

## 4. Materials and Methods

### 4.1. Ethical Approval

All animal experiments were performed according to the guidelines of the Swiss Animal Welfare Law and in compliance with the regulations of the Cantonal Veterinary Office, Zurich (Licenses for animal experimentation: ZH018/17, approved 5 May 2017; ZH146/2017, approved 3 November 2017).

### 4.2. Animal Handling

For our experiments we used *Cxcl12*-GFP mice (B6-Cxcl12-GFP<tm1Tng>) [54], kindly provided by Professor Takashi Nagasawa (Osaka University, Osaka, Japan).

The animal facility provided standardized housing conditions, with a mean room temperature of 21 ± 1 °C, relative humidity of 50% ± 5%, and 15 complete changes of filtered air per hour (HEPA H 14filter); air pressure was controlled at 50 Pa. The light/dark cycle in the animal rooms was set to a 12 h/12 h cycle (lights on at 07:00, lights off at 19:00) with artificial light of approximately 40 Lux in the cage. The animals had unrestricted access to sterilized drinking water, and ad libitum access to a pelleted and extruded mouse diet in the food hopper (Kliba No. 3436; Provimi Kliba/Granovit AG, Kaiseraugst, Switzerland). Mice were housed in a barrier-protected specific pathogen-free unit and were kept in groups of maximum five adult mice per cage in standard IVC cages (Allentown Mouse 500; 194 × 181 × 398 mm^3^, floor area 500 cm^2^; Allentown, NJ, USA) with autoclaved dust-free poplar bedding (JRS GmbH + Co KG, Rosenberg, Germany). A standard cardboard house (Ketchum Manufacturing, Brockville, ON, Canada) served as a shelter, and tissue papers were provided as nesting material. Additionally, crinklets (SAFE^®^ crinklets natural, JRS GmbH + Co KG, Rosenberg, Germany) were provided as enrichment and further nesting material. The specific pathogen-free status of the animals was monitored frequently and confirmed according to FELASA guidelines by a sentinel program. The mice were free of all viral, bacterial, and parasitic pathogens listed in FELASA recommendations [82].

Mouse pups younger than postnatal day 10 (PN10; n = 10) were sacrificed by decapitation. Mice older than postnatal day (PN15; n = 10), used for collection of dental pulp explants and isolation of dental pulp cells, were sacrificed by inhalation anesthesia (Isoflurane) followed by exposure to CO_2_ and decapitation. PN20 and older mice (n = 5), used for immunohistochemistry and immunofluorescence, were anesthetized with Ketamine/Xylazine and sacrificed by intracardiac perfusion with Phosphate Buffer Saline (PBS) followed by Paraformaldehyde (PFA) 4% in PBS.

For the tooth injury experiments, 8–10 week-old mice were used. Animals were anesthetized by intraperitoneal injection of a solution consisting of Ketamine (65 mg/kg body weight) and Xylazine (13 mg/kg body weight) dissolved in 0.9% NaCl. Cavity preparations were performed at the occlusal part of the upper first molar teeth, accessing the pulp mesio-buccal horns, using a slow-speed dental drill and a round bur size 008 (Brassler, Savannah, GA, USA) irrigated with a saline solution. Upon operation, direct dental pulp capping was performed with calcium hydroxide (Dycal Cement, LD Caulk Company, Milford, DE, USA) followed by further cavity protection filling with AH Plus Root canal sealer (Dentsply, DeTrey, Konstanz, Germany). Pain management was ensured via subcutaneous injection of Temgesic (2 mg/kg body weight) 2 h before surgery, followed by post-operatory injections with 6 h intervals for a period of 24 h and completed by supplying Temgesic in drinking water for three days post-surgery. A total of three mice were analyzed.

### 4.3. Processing of Tissues

For whole mount imaging, we used dental pulp explants isolated from the incisors and first molars of the mandible of PN5 Cxcl12-GFP^tg/+^ pups. The explants were fixed in PFA 1% for 3 h and then processed for whole mount immunofluorescent staining.

For Paraffin sections, heads from perfused mice were post-fixed by overnight incubation in PFA 4% at 4 °C, washed several times and subsequently decalcified in 10% Ethylenediamine Tetraacetic Acid (1084211000, Titriplex, Merck, Zug, Switzerland) for up to 4 weeks. Specimens were then dehydrated, embedded in Paraffin and serially sectioned at 5 µm. We applied Hematoxylin-Eosin staining for the histological examination of the specimens.

For cryosections, isolated dental pulp explants were fixed in PFA 4% for 2 h, then incubated in 30% Sucrose in PBS and finally embedded in Tissue-Tek© O.C.T. (4583, Sakura Finetek Europe B.V., Alphen aan den Rijn, Netherlands). Specimens were then serially sectioned at 10 µm.

### 4.4. Whole Mount Immunofluorescent Staining

For whole mount immunofluorescent staining we first incubated the explants in Blocking Buffer consisting of 10% normal goat serum (NGS)/0.2% Triton-X100/2% Bovine Serum Albumin (BSA) in PBS for 12 h at 4 °C. Thereafter, the samples were incubated with primary antibodies that were diluted in the blocking buffer for 72 h. The rabbit pAb anti-Laminin (dilution 1:50; ab11575, Abcam, Cambridge, UK) and the goat pAb anti-GFP (dilution 1:100; ab6673, Abcam, Cambridge, UK) were used as primary antibodies. The explants were then washed and incubated for 48–72 h with fluorescent secondary antibodies diluted in Blocking Buffer. Alexa 488-conjugated donkey anti-goat (dilution 1:200; A32814, Invitrogen/Thermo Fisher Scientific, Basel, Switzerland) and Alexa 568-conjugated donkey anti-rabbit (dilution 1:200; A10042, Invitrogen/Thermo Fisher Scientific, Basel, Switzerland) were used as secondary antibodies. All samples were counterstained with DAPI (4′,6-diamidino-2-phenylindole, Sigma-Aldrich Chemie, Germany). Samples were then cleared with FocusClear© (CelExplorer Labs Co. Hsinchu Taiwan) for 24 h, imaged via confocal fluorescent microscopy (CLSM Leica SP8), and finally analyzed using Imaris 8.5.1 (Bitplane AG, Oxford Instruments, Switzerland) and Fiji/ImageJ [83].

### 4.5. Immunohistochemistry and Immunofluorescent Staining

Immunohistochemistry on paraffin sections was performed as described previously [84]. Briefly, paraffin sections were rehydrated by incubation in Xylol followed by a series of degraded ethanol solutions (from 100% to 30%) and distilled H_2_O. Endogenous peroxidase activity was inhibited by incubating the sections in a solution composed of 3% H_2_O_2_ in −20 °C methanol for 20 min. Specimens were then rehydrated and blocked with PBS supplemented with 5% Fetal Bovine Serum (FBS) before incubation with the Goat pAb anti-GFP (dilution 1:100, ab6673, Abcam, Cambridge, United Kingdom) primary antibody for 1 h at room temperature (RT). Thereafter the sections were incubated with the appropriate secondary antibody (Vector Vectastain ABC kit–PK-6105-1, Vector Laboratories LTD, Peterborough, UK), washed for three times, and finally incubated with the AEC (3-amino-9-ethylcarbazole) according to the kit instructions (AEC HRP substrate Kit–SK4200; Vector Laboratories LTD, Peterborough, UK) to reveal the GFP staining. The slides were then counterstained with Toluidine Blue, mounted with Glycergel (C0563, Agilent Technologies, Santa Clara, CA, USA), and finally imaged with a Leica DM6000 light microscope (Leica Microsystems, Schweiz AG, Heerbrugg, Switzerland).

For double-immunofluorescent staining, endogenous peroxidase inhibition was omitted. Rabbit pAb anti-Laminin (dilution 1:50; ab11575, Abcam, Cambridge, United Kingdom), goat pAb anti-GFP (dilution 1:100; ab6673, Abcam, Cambridge, United Kingdom), rabbit pAb anti-GFP (dilution 1:100; A11122, Invitrogen/Thermo Fisher Scientific, Basel, Switzerland), goat pAb anti-Cxcr4 (dilution 1:50, GTX21670, GeneTex, Irvine, CA, USA), were used as primary antibodies. Alexa 488-conjugated donkey anti-goat (dilution 1:200; A32814, Invitrogen/Thermo Fisher Scientific, Basel, Switzerland) and Alexa 568-conjugated donkey anti-rabbit (dilution 1:200; A10042, Invitrogen/Thermo Fisher Scientific, Basel, Switzerland) were used as secondary antibodies, while DAPI (4′,6-Diamidino-2-Phenylindole–D1306; Thermo Fisher) was applied for nuclear staining. Primary antibodies were applied to the sections simultaneously for 1 h at RT, and then incubated with Fluorochrome-conjugated secondary antibodies for 1 h at RT at dark. After staining, the slides were mounted in ProLong™ Diamond Antifade Mountant (P36965; Thermo Fisher Scientific, Basel, Switzerland) and imaged with a Leica SP8 Inverted Confocal Laser Scanning Microscope (Leica Microsystems-Schweiz AG, Heerbrugg, Switzerland).

Cryosections of isolated dental pulps were thawed and air-dried for 1 h, washed with PBS to remove Tissue-Tek O.C.T, and then processed for double immunofluorescent staining as described above. The following primary antibodies were used: Goat pAb anti-GFP (dilution 1:100; ab6673, Abcam, Cambridge, United Kingdom), rabbit pAb anti-GFP (dilution 1:100; A11122, Invitrogen/Thermo Fisher Scientific, Basel, Switzerland), goat pAb anti-Sca-1 (dilution 1:100; AF1226, R&D Systems, Minneapolis, MI, USA), rabbit pAb anti-NG2 (dilution 1:100; AB5320, Millipore, Schaffhausen, Switzerland), rabbit mAb anti-Neurofilament (dilution 1:100; 2837, Cell Signaling, Danvers, MA, USA), goat pAb anti-Cxcr4 (dilution 1:50, GTX21670, GeneTex, Irvine, CA, USA), and rabbit pAb anti-Notch3 [85]. As secondary antibodies were used the Alexa 488-conjugated donkey anti-goat (dilution 1:500; A32814, Invitrogen/Thermo Fisher Scientific, Basel, Switzerland), Alexa 568-conjugated donkey anti-goat (dilution 1:500; A-11057, Invitrogen/Thermo Fisher Scientific, Basel, Switzerland), and Alexa 488-conjugated donkey anti-rabbit (dilution 1:500; A-21206, Invitrogen/Thermo Fisher Scientific, Basel, Switzerland).

### 4.6. Flow Cytometry Analysis of Dental Pulps

Dental pulps from molars and incisors were harvested and extracted as described for confocal microscopy. In total, 3–5 dental pulps from the same type of teeth were pooled, washed once in PBS and centrifuged. Dental pulps were then homogenized into cell suspensions in a digestion buffer composed of Dulbecco’s modified Eagle’s medium (DMEM) GlutaMAX/10 mM HEPES/10% FCS by gentle and repeated mixing using a syringe with a 21 Gauge needle. For enzymatic digestion a mixture of DNase (final concentration 0.2 mg/mL) and Collagenase Type2 (0.04 g/mL) was added to the media, and cell suspensions were incubated at 37 °C for 45 min under gentle rocking. Digestion was inactivated by addition of cold PBS, and cell suspensions were filtered through a 70 µm cell strainer. Cells were then centrifuged, resuspended in PBS, blocked using TruStain fcX (BioLegend, San Diego, USA) and successively stained with cocktails of fluorescently labeled antibodies [22]. After immunostaining, cells were washed twice in PBS, resuspended in PBS/10% FCS and analyzed on an LSR II Fortessa (BD Biosciences, San Jose, CA, USA). Data analysis was performed using the FlowJo 10 software package.

### 4.7. Cxcl12-GFP^+^ Dental Pulp Cells Culture

Cxcl12-GFP^+^ dental pulp cells were isolated from molars and incisors of PN15 Cxcl12-GFP mice. Dental pulps were minced to small pieces and digested in Collagenase P (2.5 mg/mL) for 30 min at 37 °C. Cell suspensions were then filtered through a 70 μm cell strainer. Sorted Cxcl12-GFP^+^ dental pulp cells were cultured in DMEM supplemented with 30% FBS. Osteogenic differentiation was induced by culturing the cells in DMEM supplemented with 10% FBS, Ascorbic Acid (200 μM), β-Glycerolphosphate (10 mM), Dexamethasone (10 nM) (Sigma-Aldrich/Merck, Darmstadt, Germany), and Amphotericin B (0.25 μg/μL) (ThermoFisher Scientific, Switzerland). After 10 days of treatment, cells were harvested and snap frozen in liquid nitrogen. To inhibit the Notch signaling pathway, Cxcl12-GFP^+^ dental pulp cells were cultured in DMEM supplemented with 30% FBS and 2.5 μM DAPT (*N*-[*N*-(3,5-Difluorophenacetyl)-*L*-alanyl]-S-phenylglycine t-butyl ester; D5942, Sigma-Aldrich, Buchs, Switzerland). After 48 h of treatment, cells were harvested and snap frozen in liquid nitrogen.

### 4.8. Real-Time PCR

RNA was isolated from snap-frozen cells using the RNeasy Plus Universal Mini Kit (Qiagen AG, Hombrechtikon ZH, Switzerland). Reverse transcription of the isolated RNA was performed using the iScript™ cDNA synthesis Kit and according to the instructions given (Bio-Rad Laboratories AG, Cressier FR, Switzerland). Briefly, 1000 ng of RNA were used for reverse transcription into cDNA. Nuclease-free water was added to add up to a total of 15 μL. In total, 4 μL of 5xiScript reaction mix and 1 μL of iScript reverse transcriptase were added per sample in order to obtain a total volume of 20 μL. The reaction mix was then incubated for 5 min at 25 °C, for 30 min at 42 °C and for 5 min at 85 °C using a Biometra TPersonal Thermocycler (Biometra AG, Göttingen, Germany). The 3-step quantitative real-time PCRs were performed using an Eco Real-Time PCR System (Illumina Inc., San Diego CA, USA). Expression level analysis of 36b4 (housekeeping gene), Notch3, CD90/Thy1, Runx2, Osx, Pparg, Plp1, Sox9, Mdk, CD45/PTPRC, Dspp, PECAM1/CD31, and Cxcl12 were carried out using the SYBR^®^ Green PCR Master Mix (Applied Biosystems, Carlsbad CA, USA) in combination with oligonucleotide primers. The thermocycling conditions were: 95°C for 10 min, followed by 45 cycles of 95 °C for 15 s, 55 °C for 30 s and 60 °C for 1 min. Melt curve analysis was performed at 95 °C for 15 s, 50 °C for 15 s and 95 °C for 15 s. Expression levels were normalized to the Ct-value of the 36b4 housekeeping gene. Gene expression analysis was performed on three independent samples per condition.

## Supplementary Materials

The following are available online at https://www.mdpi.com/1422-0067/22/6/3024/s1.

## References

[1] Mitsiadis T.A., Graf D. (2009). Cell fate determination during tooth development and regeneration. Birth Defects Res. Part C Embryo Today.

[2] Nanci A., Nanci A. (2013). Ten Cate’s Oral Histology.

[3] Smith P.C., Martinez C., Martinez J., McCulloch C.A. (2019). Role of Fibroblast Populations in Periodontal Wound Healing and Tissue Remodeling. Front. Physiol..

[4] Sodek J., Overall C.M. (1992). Matrix metalloproteinases in periodontal tissue remodelling. Matrix Suppl..

[5] Pagella P., Neto E., Lamghari M., Mitsiadis T.A. (2015). Investigation of orofacial stem cell niches and their innervation through microfluidic devices. Eur. Cell Mater..

[6] Orsini G., Pagella P., Mitsiadis T.A. (2018). Modern Trends in Dental Medicine: An Update for Internists. Am. J. Med..

[7] Tomokiyo A., Wada N., Maeda H. (2019). Periodontal Ligament Stem Cells: Regenerative Potency in Periodontium. Stem Cells Dev..

[8] Trubiani O., Pizzicannella J., Caputi S., Marchisio M., Mazzon E., Paganelli R., Paganelli A., Diomede F. (2019). Periodontal Ligament Stem Cells: Current Knowledge and Future Perspectives. Stem Cells Dev..

[9] Chen H., Fu H., Wu X., Duan Y., Zhang S., Hu H., Liao Y., Wang T., Yang Y., Chen G. (2020). Regeneration of pulpo-dentinal-like complex by a group of unique multipotent CD24a^+^ stem cells. Sci. Adv..

[10] Shi X., Mao J., Liu Y. (2020). Pulp stem cells derived from human permanent and deciduous teeth: Biological characteristics and therapeutic applications. Stem Cells Transl. Med..

[11] Shah D., Lynd T., Ho D., Chen J., Vines J., Jung H.D., Kim J.H., Zhang P., Wu H., Jun H.W. (2020). Pulp-Dentin Tissue Healing Response: A Discussion of Current Biomedical Approaches. J. Clin. Med..

[12] Xuan K., Li B., Guo H., Sun W., Kou X., He X., Zhang Y., Sun J., Liu A., Liao L. (2018). Deciduous autologous tooth stem cells regenerate dental pulp after implantation into injured teeth. Sci. Transl. Med..

[13] Scadden D.T. (2014). Nice neighborhood: Emerging concepts of the stem cell niche. Cell.

[14] Lane S.W., Williams D.A., Watt F.M. (2014). Modulating the stem cell niche for tissue regeneration. Nat. Biotechnol..

[15] Janssens R., Struyf S., Proost P. (2018). The unique structural and functional features of CXCL12. Cell Mol. Immunol..

[16] Zhu C., Yao W.L., Tan W., Zhang C.H. (2017). SDF-1 and CXCR4 play an important role in adult SVZ lineage cell proliferation and differentiation. Brain Res..

[17] Greenbaum A., Hsu Y.M., Day R.B., Schuettpelz L.G., Christopher M.J., Borgerding J.N., Nagasawa T., Link D.C. (2013). CXCL12 in early mesenchymal progenitors is required for haematopoietic stem-cell maintenance. Nature.

[18] Karin N. (2010). The multiple faces of CXCL12 (SDF-1α) in the regulation of immunity during health and disease. J. Leukoc. Biol..

[19] Kitaori T., Ito H., Schwarz E.M., Tsutsumi R., Yoshitomi H., Oishi S., Nakano M., Fujii N., Nagasawa T., Nakamura T. (2009). Stromal cell-derived factor 1/CXCR4 signaling is critical for the recruitment of mesenchymal stem cells to the fracture site during skeletal repair in a mouse model. Arthritis Rheum..

[20] Abbott J.D., Huang Y., Liu D., Hickey R., Krause D.S., Giordano F.J. (2004). Stromal cell-derived factor-1α plays a critical role in stem cell recruitment to the heart after myocardial infarction but is not sufficient to induce homing in the absence of injury. Circulation.

[21] Sugiyama T., Kohara H., Noda M., Nagasawa T. (2006). Maintenance of the hematopoietic stem cell pool by CXCL12-CXCR4 chemokine signaling in bone marrow stromal cell niches. Immunity.

[22] Gomariz A., Helbling P.M., Isringhausen S., Suessbier U., Becker A., Boss A., Nagasawa T., Paul G., Goksel O., Szekely G. (2018). Quantitative spatial analysis of haematopoiesis-regulating stromal cells in the bone marrow microenvironment by 3D microscopy. Nat. Commun..

[23] Addington C.P., Pauken C.M., Caplan M.R., Stabenfeldt S.E. (2014). The role of SDF-1alpha-ECM crosstalk in determining neural stem cell fate. Biomaterials.

[24] Busillo J.M., Benovic J.L. (2007). Regulation of CXCR4 signaling. Biochim. Biophys. Acta.

[25] Kawaguchi N., Zhang T.T., Nakanishi T. (2019). Involvement of CXCR4 in Normal and Abnormal Development. Cells.

[26] Ma Q., Jones D., Borghesani P.R., Segal R.A., Nagasawa T., Kishimoto T., Bronson R.T., Springer T.A. (1998). Impaired B-lymphopoiesis, myelopoiesis, and derailed cerebellar neuron migration in CXCR4- and SDF-1-deficient mice. Proc. Natl. Acad. Sci. USA.

[27] Escot S., Blavet C., Faure E., Zaffran S., Duband J.L., Fournier-Thibault C. (2016). Disruption of CXCR4 signaling in pharyngeal neural crest cells causes DiGeorge syndrome-like malformations. Development.

[28] Ding B.S., Cao Z., Lis R., Nolan D.J., Guo P., Simons M., Penfold M.E., Shido K., Rabbany S.Y., Rafii S. (2014). Divergent angiocrine signals from vascular niche balance liver regeneration and fibrosis. Nature.

[29] Zhang M., Mal N., Kiedrowski M., Chacko M., Askari A.T., Popovic Z.B., Koc O.N., Penn M.S. (2007). SDF-1 expression by mesenchymal stem cells results in trophic support of cardiac myocytes after myocardial infarction. FASEB J..

[30] Matsushita Y., Nagata M., Kozloff K.M., Welch J.D., Mizuhashi K., Tokavanich N., Hallett S.A., Link D.C., Nagasawa T., Ono W. (2020). A Wnt-mediated transformation of the bone marrow stromal cell identity orchestrates skeletal regeneration. Nat. Commun..

[31] Jiang H.W., Ling J.Q., Gong Q.M. (2008). The expression of stromal cell-derived factor 1 (SDF-1) in inflamed human dental pulp. J. Endod..

[32] Yang S., Edman L.C., Sanchez-Alcaniz J.A., Fritz N., Bonilla S., Hecht J., Uhlen P., Pleasure S.J., Villaescusa J.C., Marin O. (2013). Cxcl12/Cxcr4 signaling controls the migration and process orientation of A9–A10 dopaminergic neurons. Development.

[33] Lieberam I., Agalliu D., Nagasawa T., Ericson J., Jessell T.M. (2005). A Cxcl12-CXCR4 chemokine signaling pathway defines the initial trajectory of mammalian motor axons. Neuron.

[34] Zanetti G., Negro S., Megighian A., Mattarei A., Lista F., Fillo S., Rigoni M., Pirazzini M., Montecucco C. (2019). A CXCR4 receptor agonist strongly stimulates axonal regeneration after damage. Ann. Clin. Transl. Neurol..

[35] Petit I., Jin D., Rafii S. (2007). The SDF-1-CXCR4 signaling pathway: A molecular hub modulating neo-angiogenesis. Trends Immunol..

[36] Rafii S., Cao Z., Lis R., Siempos I.I., Chavez D., Shido K., Rabbany S.Y., Ding B.S. (2015). Platelet-derived SDF-1 primes the pulmonary capillary vascular niche to drive lung alveolar regeneration. Nat. Cell Biol..

[37] Takabatake Y., Sugiyama T., Kohara H., Matsusaka T., Kurihara H., Koni P.A., Nagasawa Y., Hamano T., Matsui I., Kawada N. (2009). The CXCL12 (SDF-1)/CXCR4 axis is essential for the development of renal vasculature. J. Am. Soc. Nephrol..

[38] Jiang L., Zhu Y.Q., Du R., Gu Y.X., Xia L., Qin F., Ritchie H.H. (2008). The expression and role of stromal cell-derived factor-1α-CXCR4 axis in human dental pulp. J. Endod..

[39] Kaku M., Kitami M., Rocabado J.M.R., Ida T., Akiba Y., Uoshima K. (2017). Recruitment of bone marrow-derived cells to the periodontal ligament via the stromal cell-derived factor-1/C-X-C chemokine receptor type 4 axis. J. Periodontal Res..

[40] Li M., Sun X., Ma L., Jin L., Zhang W., Xiao M., Yu Q. (2017). SDF-1/CXCR4 axis induces human dental pulp stem cell migration through FAK/PI3K/Akt and GSK3β/β-catenin pathways. Sci. Rep..

[41] Liu J.Y., Chen X., Yue L., Huang G.T., Zou X.Y. (2015). CXC Chemokine Receptor 4 Is Expressed Paravascularly in Apical Papilla and Coordinates with Stromal Cell-derived Factor-1α during Transmigration of Stem Cells from Apical Papilla. J. Endod..

[42] Akazawa Y., Hasegawa T., Yoshimura Y., Chosa N., Asakawa T., Ueda K., Sugimoto A., Kitamura T., Nakagawa H., Ishisaki A. (2015). Recruitment of mesenchymal stem cells by stromal cell-derived factor 1α in pulp cells from deciduous teeth. Int. J. Mol. Med..

[43] Suzuki T., Lee C.H., Chen M., Zhao W., Fu S.Y., Qi J.J., Chotkowski G., Eisig S.B., Wong A., Mao J.J. (2011). Induced migration of dental pulp stem cells for in vivo pulp regeneration. J. Dent. Res..

[44] Yang J.W., Zhang Y.F., Wan C.Y., Sun Z.Y., Nie S., Jian S.J., Zhang L., Song G.T., Chen Z. (2015). Autophagy in SDF-1α-mediated DPSC migration and pulp regeneration. Biomaterials.

[45] Xu M., Wei X., Fang J., Xiao L. (2019). Combination of SDF-1 and bFGF promotes bone marrow stem cell-mediated periodontal ligament regeneration. Biosci. Rep..

[46] Cavalla F., Reyes M., Vernal R., Alvarez C., Paredes R., Garcia-Sesnich J., Infante M., Farina V., Barron I., Hernandez M. (2013). High levels of CXC ligand 12/stromal cell-derived factor 1 in apical lesions of endodontic origin associated with mast cell infiltration. J. Endod..

[47] Iohara K., Imabayashi K., Ishizaka R., Watanabe A., Nabekura J., Ito M., Matsushita K., Nakamura H., Nakashima M. (2011). Complete pulp regeneration after pulpectomy by transplantation of CD105^+^ stem cells with stromal cell-derived factor-1. Tissue Eng. Part A.

[48] Lovschall H., Mitsiadis T.A., Poulsen K., Jensen K.H., Kjeldsen A.L. (2007). Coexpression of Notch3 and Rgs5 in the pericyte-vascular smooth muscle cell axis in response to pulp injury. Int. J. Dev. Biol..

[49] Crisan M., Yap S., Casteilla L., Chen C.W., Corselli M., Park T.S., Andriolo G., Sun B., Zheng B., Zhang L. (2008). A perivascular origin for mesenchymal stem cells in multiple human organs. Cell Stem Cell.

[50] Mitsiadis T.A., Muramatsu T., Muramatsu H., Thesleff I. (1995). Midkine (MK), a heparin-binding growth/differentiation factor, is regulated by retinoic acid and epithelial-mesenchymal interactions in the developing mouse tooth, and affects cell proliferation and morphogenesis. J. Cell Biol..

[51] Mitsiadis T.A., Caton J., de Bari C., Bluteau G. (2008). The large functional spectrum of the heparin-binding cytokines MK and HB-GAM in continuously growing organs: The rodent incisor as a model. Dev. Biol..

[52] Ie M.S., Wang T.L. (2007). Notch signaling, gamma-secretase inhibitors, and cancer therapy. Cancer Res..

[53] Lapidot T., Dar A., Kollet O. (2005). How do stem cells find their way home?. Blood.

[54] Ara T., Tokoyoda K., Sugiyama T., Egawa T., Kawabata K., Nagasawa T. (2003). Long-term hematopoietic stem cells require stromal cell-derived factor-1 for colonizing bone marrow during ontogeny. Immunity.

[55] Mitsiadis T.A., Caton J., Pagella P., Orsini G., Jimenez-Rojo L. (2017). Monitoring Notch Signaling-Associated Activation of Stem Cell Niches within Injured Dental Pulp. Front. Physiol..

[56] Shi S., Gronthos S. (2003). Perivascular niche of postnatal mesenchymal stem cells in human bone marrow and dental pulp. J. Bone Miner. Res..

[57] Vidovic I., Banerjee A., Fatahi R., Matthews B.G., Dyment N.A., Kalajzic I., Mina M. (2017). αSMA-Expressing Perivascular Cells Represent Dental Pulp Progenitors In Vivo. J. Dent. Res..

[58] Feng J., Mantesso A., de Bari C., Nishiyama A., Sharpe P.T. (2011). Dual origin of mesenchymal stem cells contributing to organ growth and repair. Proc. Natl. Acad. Sci. USA.

[59] Kaukua N., Shahidi M.K., Konstantinidou C., Dyachuk V., Kaucka M., Furlan A., An Z., Wang L., Hultman I., Ahrlund-Richter L. (2014). Glial origin of mesenchymal stem cells in a tooth model system. Nature.

[60] Harada H., Kettunen P., Jung H.S., Mustonen T., Wang Y.A., Thesleff I. (1999). Localization of putative stem cells in dental epithelium and their association with Notch and FGF signaling. J. Cell Biol..

[61] Mitsiadis T.A., Barrandon O., Rochat A., Barrandon Y., de Bari C. (2007). Stem cell niches in mammals. Exp. Cell Res..

[62] Rafii S., Butler J.M., Ding B.S. (2016). Angiocrine functions of organ-specific endothelial cells. Nature.

[63] Agarwal P., Isringhausen S., Li H., Paterson A.J., He J., Gomariz A., Nagasawa T., Nombela-Arrieta C., Bhatia R. (2019). Mesenchymal Niche-Specific Expression of Cxcl12 Controls Quiescence of Treatment-Resistant Leukemia Stem Cells. Cell Stem Cell.

[64] Potten C.S., Loeffler M. (1990). Stem cells: Attributes, cycles, spirals, pitfalls and uncertainties. Lessons for and from the crypt. Development.

[65] Artavanis-Tsakonas S., Muskavitch M.A. (2010). Notch: The past, the present, and the future. Curr. Top. Dev. Biol..

[66] Pang Y.W., Feng J., Daltoe F., Fatscher R., Gentleman E., Gentleman M.M., Sharpe P.T. (2016). Perivascular Stem Cells at the Tip of Mouse Incisors Regulate Tissue Regeneration. J. Bone Miner. Res..

[67] Blache U., Vallmajo-Martin Q., Horton E.R., Guerrero J., Djonov V., Scherberich A., Erler J.T., Martin I., Snedeker J.G., Milleret V. (2018). Notch-inducing hydrogels reveal a perivascular switch of mesenchymal stem cell fate. EMBO Rep..

[68] Xiao M., Qiu J., Kuang R., Zhang B., Wang W., Yu Q. (2019). Synergistic effects of stromal cell-derived factor-1α and bone morphogenetic protein-2 treatment on odontogenic differentiation of human stem cells from apical papilla cultured in the VitroGel 3D system. Cell Tissue Res..

[69] Kim D.S., Kim Y.S., Bae W.J., Lee H.J., Chang S.W., Kim W.S., Kim E.C. (2014). The role of SDF-1 and CXCR4 on odontoblastic differentiation in human dental pulp cells. Int. Endod. J..

[70] Mitsiadis T.A., Orsini G., Jimenez-Rojo L. (2015). Stem cell-based approaches in dentistry. Eur. Cell Mater..

[71] Mitsiadis T.A., Rahiotis C. (2004). Parallels between tooth development and repair: Conserved molecular mechanisms following carious and dental injury. J. Dent. Res..

[72] Theiss H.D., Vallaster M., Rischpler C., Krieg L., Zaruba M.M., Brunner S., Vanchev Y., Fischer R., Gröbner M., Huber B. (2011). Dual stem cell therapy after myocardial infarction acts specifically by enhanced homing via the SDF-1/CXCR4 axis. Stem Cell Res..

[73] Yu J., Li M., Qu Z., Yan D., Li D., Ruan Q. (2010). SDF-1/CXCR4-mediated migration of transplanted bone marrow stromal cells toward areas of heart myocardial infarction through activation of PI3K/Akt. J. Cardiovasc. Pharmacol..

[74] Nakashima K., Zhou X., Kunkel G., Zhang Z., Deng J.M., Behringer R.R., de Crombrugghe B. (2002). The novel zinc finger-containing transcription factor osterix is required for osteoblast differentiation and bone formation. Cell.

[75] Bruderer M., Richards R.G., Alini M., Stoddart M.J. (2014). Role and regulation of RUNX2 in osteogenesis. Eur. Cell Mater..

[76] Balic A., Aguila H.L., Caimano M.J., Francone V.P., Mina M. (2010). Characterization of stem and progenitor cells in the dental pulp of erupted and unerupted murine molars. Bone.

[77] Zhang C., Chang J., Sonoyama W., Shi S., Wang C.Y. (2008). Inhibition of human dental pulp stem cell differentiation by Notch signaling. J. Dent. Res..

[78] Kimura Y., Komaki M., Iwasaki K., Sata M., Izumi Y., Morita I. (2014). Recruitment of bone marrow-derived cells to periodontal tissue defects. Front. Cell Dev. Biol..

[79] Zhou J., Shi S., Shi Y., Xie H., Chen L., He Y., Guo W., Wen L., Jin Y. (2011). Role of bone marrow-derived progenitor cells in the maintenance and regeneration of dental mesenchymal tissues. J. Cell Physiol..

[80] Hatano K., Ishida Y., Yamaguchi H., Hosomichi J., Suzuki J.I., Usumi-Fujita R., Shimizu Y., Shibutani N., Kaneko S., Ono T. (2018). The chemokine receptor type 4 antagonist, AMD3100, interrupts experimental tooth movement in rats. Arch. Oral. Biol..

[81] Zhang L., Zhou Y., Sun X., Zhou J., Yang P. (2017). CXCL12 overexpression promotes the angiogenesis potential of periodontal ligament stem cells. Sci. Rep..

[82] Convenor M.M., Berard M., Feinstein R., Gallagher A., Illgen-Wilcke B., Pritchett-Corning K., Raspa M. (2014). FELASA recommendations for the health monitoring of mouse, rat, hamster, guinea pig and rabbit colonies in breeding and experimental units. Lab. Anim..

[83] Schindelin J., Arganda-Carreras I., Frise E., Kaynig V., Longair M., Pietzsch T., Preibisch S., Rueden C., Saalfeld S., Schmid B. (2012). Fiji: An open-source platform for biological-image analysis. Nat. Methods.

[84] Mitsiadis T.A., Salmivirta M., Muramatsu T., Muramatsu H., Rauvala H., Lehtonen E., Jalkanen M., Thesleff I. (1995). Expression of the heparin-binding cytokines, midkine (MK) and HB-GAM (pleiotrophin) is associated with epithelial-mesenchymal interactions during fetal development and organogenesis. Development.

[85] Mitsiadis T.A., Lardelli M., Lendahl U., Thesleff I. (1995). Expression of Notch 1, 2 and 3 is regulated by epithelial-mesenchymal interactions and retinoic acid in the developing mouse tooth and associated with determination of ameloblast cell fate. J. Cell Biol..

