# Supplementary material for: Distinct Expression Patterns of Cxcl12 in Mesenchymal Stem Cell Niches of Intact and Injured Rodent Teeth
Source: Int J Mol Sci. 2021 Mar 16;22(6):3024. doi: 10.3390/ijms22063024 (PMC8002260; doi:10.3390/ijms22063024)
Supplement: Supplementary file 1 [file ijms-22-03024-s001.pdf]

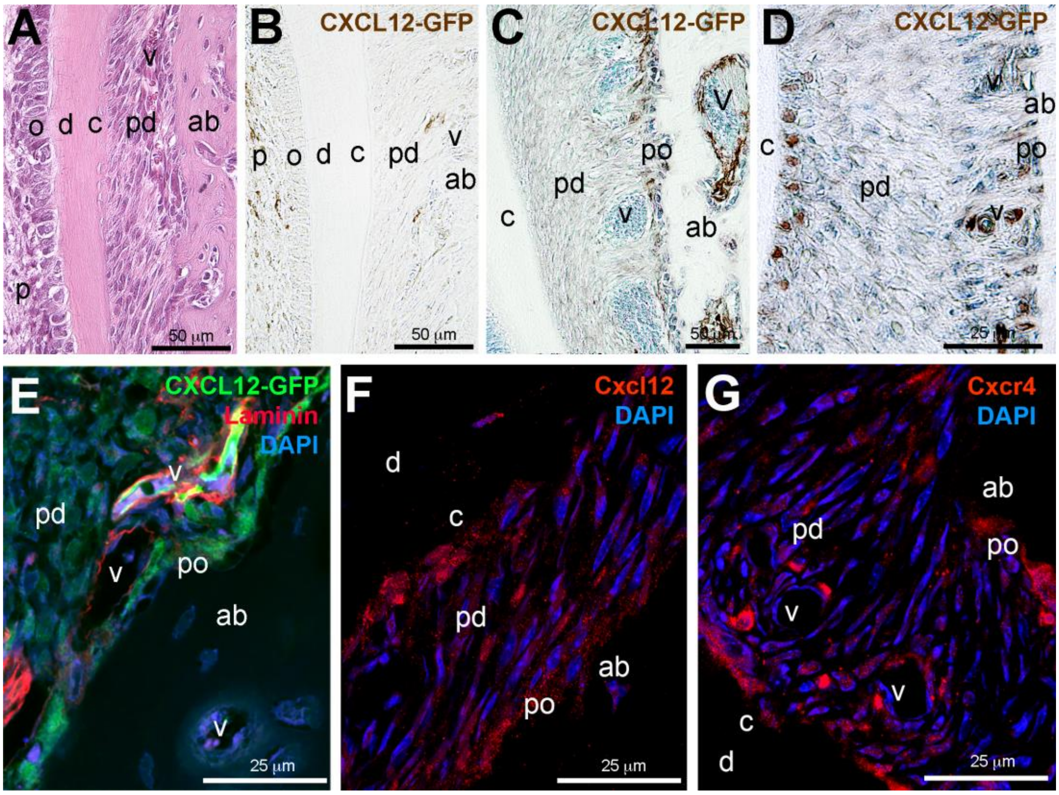

Figure 1. Expression of Cxcl12 and Cxcr4 in the periodontium. **A)** Histology of the mouse first molar periodontium upon staining with haematoxylin-eosin. **B)** Immunohistochemistry showing *Cxcl12*-GFP expression (brown colour) in the periodontium of mouse first molars. **C, D)** Immunohistochemistry showing *Cxcl12*-GFP expression (brown colour) in the periodontium of mouse lower incisors. **E)** Double immunofluorescent staining showing expression of *Cxcl12*-GFP (green colour) relative to Laminin (red colour) in the periodontium of mouse first molars. DAPI in blue colour. **F)** Immunofluorescent staining showing localization of the Cxcl12 protein in the molar periodontium. DAPI in blue colour. **G)** Immunofluorescent staining showing localization of the Cxcr4 protein in the molar periodontium. DAPI in blue colour. Abbreviations: ab, alveolar bone; c, cementum; d, dentin; o, odontoblasts; p, dental pulp; pd, periodontium; po, periosteum; v, vessel. Scale bars: A-C, 50 µm; D-G, 25 µm.
